# Mathematics of biomedical imaging today—a perspective

**DOI:** 10.1088/2516-1091/acd973

**Published:** 2023-08-04

**Authors:** Marta M Betcke, Carola-Bibiane Schönlieb

**Affiliations:** 1 Department of Computer Science, University College London, London WC1E6BT, United Kingdom; 2 Department for Applied Mathematics and Theoretical Physics, University of Cambridge, Cambridge, United Kingdom

**Keywords:** imaging, biomedicine, mathematics

## Abstract

Biomedical imaging is a fascinating, rich and dynamic research area, which has huge importance in biomedical research and clinical practice alike. The key technology behind the processing, and automated analysis and quantification of imaging data is mathematics. Starting with the optimisation of the image acquisition and the reconstruction of an image from indirect tomographic measurement data, all the way to the automated segmentation of tumours in medical images and the design of optimal treatment plans based on image biomarkers, mathematics appears in all of these in different flavours. Non-smooth optimisation in the context of sparsity-promoting image priors, partial differential equations for image registration and motion estimation, and deep neural networks for image segmentation, to name just a few. In this article, we present and review mathematical topics that arise within the whole biomedical imaging pipeline, from tomographic measurements to clinical support tools, and highlight some modern topics and open problems. The article is addressed to both biomedical researchers who want to get a taste of where mathematics arises in biomedical imaging as well as mathematicians who are interested in what mathematical challenges biomedical imaging research entails.

## 1 Biomedical imaging

Vision being the primary human sense, it is no surprise that an image became a preferable way of representing spatially varying information and medical imaging is no exception. Indeed, biomedical imaging is the main mean for us to look into the human body in a non-invasive way. What we see ranges from the very small to the very big. From electron tomography for analysing atomic structures of molecules on lengthscales of a couple of Angstroems [76], to light-microscopy for tissue and cell analysis [79, 89], to x-ray and computed tomography (CT) [75], and molecular imaging such as magnetic resonance imaging (MRI) [7] and positron emission tomography (PET) [90], to wave-imaging such as ultrasound [94], to optical and photo-acoustic tomographic (PAT) imaging techniques [5, 9]. The main use-cases for medical imaging are

(i) *medical research*: where images are used to investigate diseases and derive novel biomarkers for cancer, cardiovascular diseases, cognitive decline, etc through cohort analysis and individual stratification;
(ii) *clinical practice*: where medical imaging is used for preventive screening, diagnosis, treatment planning and image-guided clinical interventions.

The trend is clear. The variety of image data that is being collected at increasing spatial and temporal scales clearly goes beyond capabilities of manual interpretation. Automated algorithms (AI techniques) are needed to interpret these images, and must be developed taking into account the four Vs of big data (quoting IBM’s categorisation^3^). The first V is volume, with the increasing resolution and dimensionality of images as hardware is improving and affordable emerging imaging techniques make their way into the clinic. The second V is variety, spanning various imaging modalities mentioned before. The third is velocity, the steadily increasing speed and rate at which the data is acquired, enables new applications (such as e.g. imaging of change) as well as more comprehensive screening. The final V is veracity, considering uncertainty, noise and artefacts in the image data. The modern day biomedical imaging pathway navigates the process from data acquisition (measurements), through to the formation of an image (image reconstruction), the extraction of biomarkers (also called image analysis or image quantification), and eventually image-based statistics for prediction and further analysis. With digital images basically just being a bunch of numbers stored in a possibly high-dimensional vector, each of these processing and analysis steps has mathematics at its core, each in its different way. The mathematics involved with image data is challenging as image sizes and properties vary, hence require bespoke mathematics designed for the particular data-type, and because image data is big with biomedical images easily featuring a million unknowns.

### 1.1 Outline

**Figure 1:**
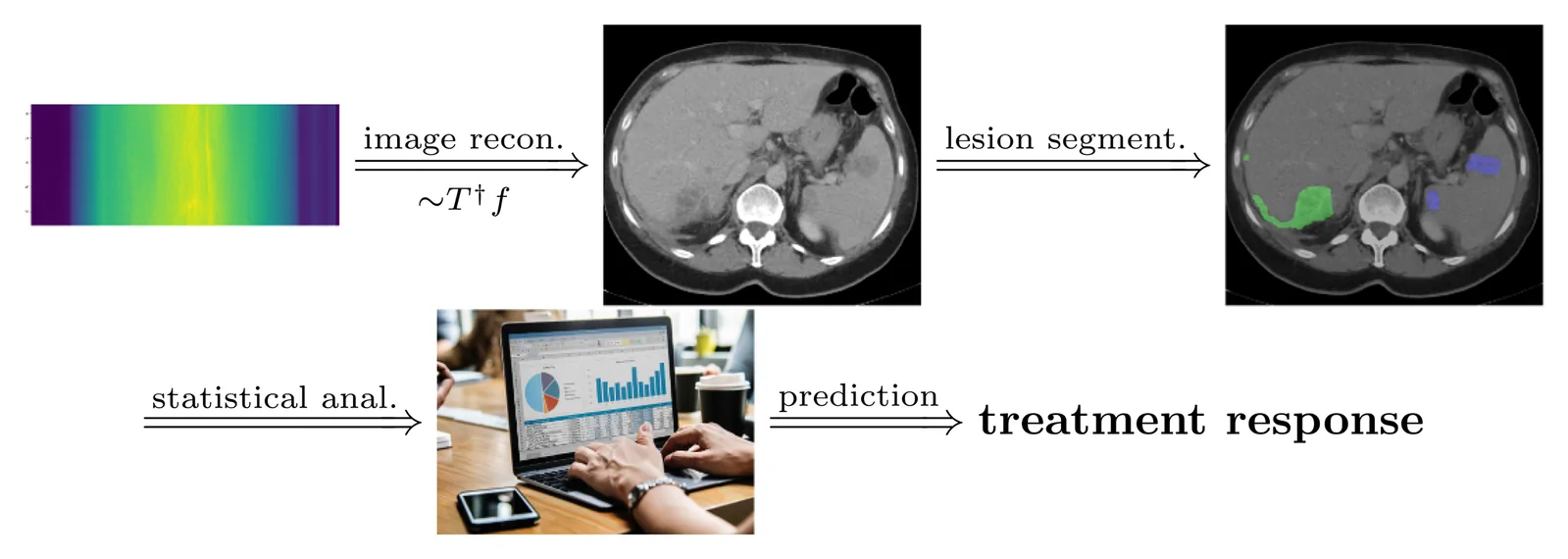
Exemplar biomedical imaging pathway. From left to right: One CT projection measurement; 2D slice of reconstructed 3D CT volume; segmentation of cancerous lesions (different colours correspond to different tumour types); statistical analysis which could include image features such as size and shape parameters of segmented lesions, texture features within lesions, etc; and finally the prediction based on the statistical analysis which could for instance be response to chemotherapy treatment. CT data and segmentation are courtesy of Evis Sala and Romana Woitek. Parts of this image have been obtained by the authors from the Pexels website where it was made available under the Pexels License. It is included within this article on that basis.

In what follows, we will investigate each step of the biomedical imaging pathway figure 1 and discuss its mathematical challenges in sections 2–5. In sections 6–8 we pick out and discuss in more detail three modern topics in biomedical imaging which depict the recent trend in bioimaging of intra- and inter- stage task fusion, as well as deep learning as a powerful mean to model images and their information content. We close in section 9 with a short discussion of how mathematics can continue to aid imaging in particular in the advent of AI techniques making their way into healthcare.

With this perspectives piece we aim to address both biomedical researchers with a keen interest to find out where mathematics enters biomedical imaging research and development, and mathematicians who want to get a flavour for the kind of mathematical problems that appear in biomedical imaging. To serve both audiences, our discussion is a mix of conceptual explanations that include some name dropping and references for further reading for the mathematically interested reader, and a more direct exemplified mathematical description for the modern research topics discussions in sections 6–8.

## 2 How to image

Every biomedical imaging task initiates with a question: what do we want to observe, what qualities of the image do we require and how to acquire such image. This is possibly the most interdisciplinary task of the biomedical imaging framework as it requires clinical understanding of the condition/pathology, expertise in the underlying biological processes and interaction of the imaging agent with the biology of the object, knowledge of the physics of the deployed modality to model the image formation process, engineering skills to construct a device and finally mathematics to formulate, analyse and solve the image reconstruction problem. In practice, we do not have the luxury to go through this process on a task by task bases, but groups of tasks are targeted and new tasks are continuously identified as the technology evolves. As a result most of the time we consider available modalities first and attempt to compensate for their limitations with adjusted imaging protocols, combining different technologies and mathematics.

**Figure 2:**
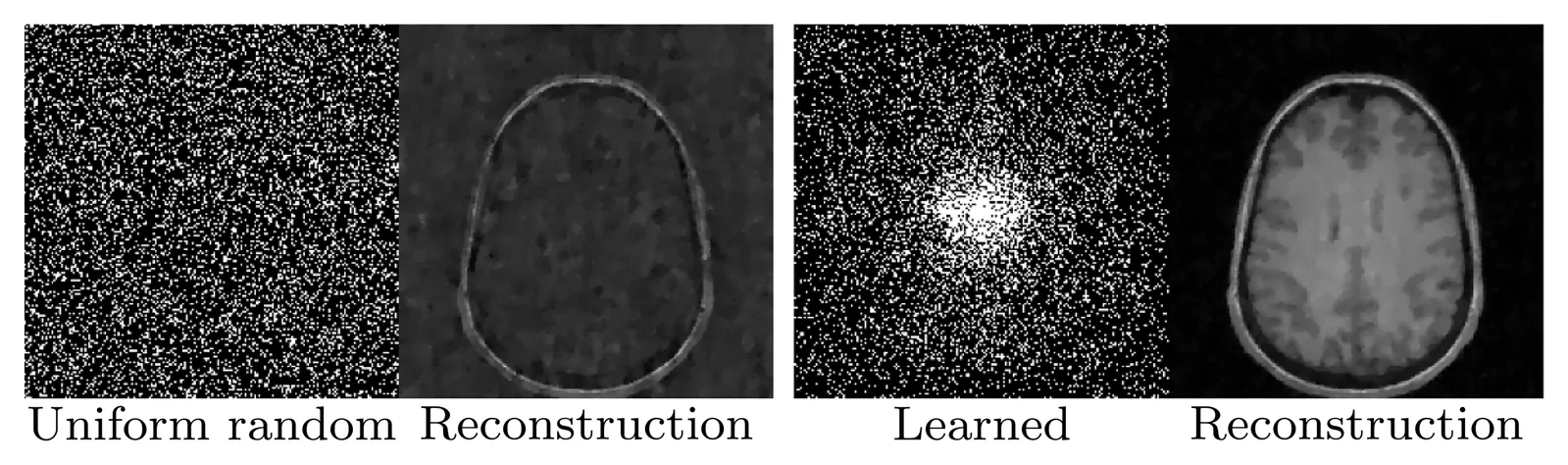
The importance of a good choice of sampling pattern in Fourier space (fltr): uniform random pattern as suggested by the classical compressed sensing literature [29] and total variation reconstruction (cf section 6) on a test image, and an equally sparse pattern learned with the algorithm in [91] and visually similar to sampling patterns coming out of a revised compressed sensing theory [1] and total variation reconstruction for the same test image.

The choice of the technology is usually based on various factors:

(i) Individual features of different imaging techniques, such as CT or MRI for visualising anatomical structures, PET for metabolic activity, and microscopy for small tissue sample imaging of biopsies. Often, more than one imaging modality is used in medical research and clinical practice. Multi-modality imaging is a major topic in mathematical imaging, where it gives rise to new ways of image reconstruction (see also section 6) with research focussing on proposal of new image priors (regularisers) that model correlations between the different modalities [47, 48], challenges around image fusion with new models that are based on image registration [71], deconvolution and interpolation [24], and further analysis of these fused images based on high-dimensional statistics, functional data analysis and machine learning [23, 82] (compare also to the discussion of image biomarkers in section 4).
(ii) Trade-off between availability and cost of an imaging technique—e.g. X-ray and ultrasound imaging are widely-spread imaging techniques that can be found in every clinic and even some GP or specialist medical practices, while MRI is expensive and usually only available in larger clinics.
(iii) Medical imaging is complicated by the fact that the resolution of the image does not only depend on the hardware but also on the acquisition time and radiation dose. The challenge here is to produce a high quality description of patients and their ailments from data which is necessarily limited by the capability of scanners and the need to image fast, and minimise exposure to harmful radiation. In MRI, for example, the measurements are—roughly speaking—Fourier coefficients of the image intensity function that represents the anatomical structure of the imaged body part. One can imagine now, that the resolution of the image (number of unknowns representing the image intensity function) is correlated with the amount of Fourier samples acquired by the machine. Acquiring Fourier samples, however, takes time, as the measurement device needs to excite the water molecules in different ways to trigger different frequency responses [7]. The discovery of compressed sensing [29, 45], i.e. the nonadaptive compressed acquisition of data, gave rise to efficient numerical algorithms which allow one to sense/acquire only the essential features of signals, and in turn to recover them by very sparse sampling of Fourier coefficients in MRI [68]. The latter also naturally led to research into where best to sample in Fourier space with both theoretical contributions in applied harmonic analysis [1, 18, 63] and practical solutions in applied analysis and optimisation [20, 36, 91], cf also figure 2.

## 3 From measurements to an image

**Figure 3:**
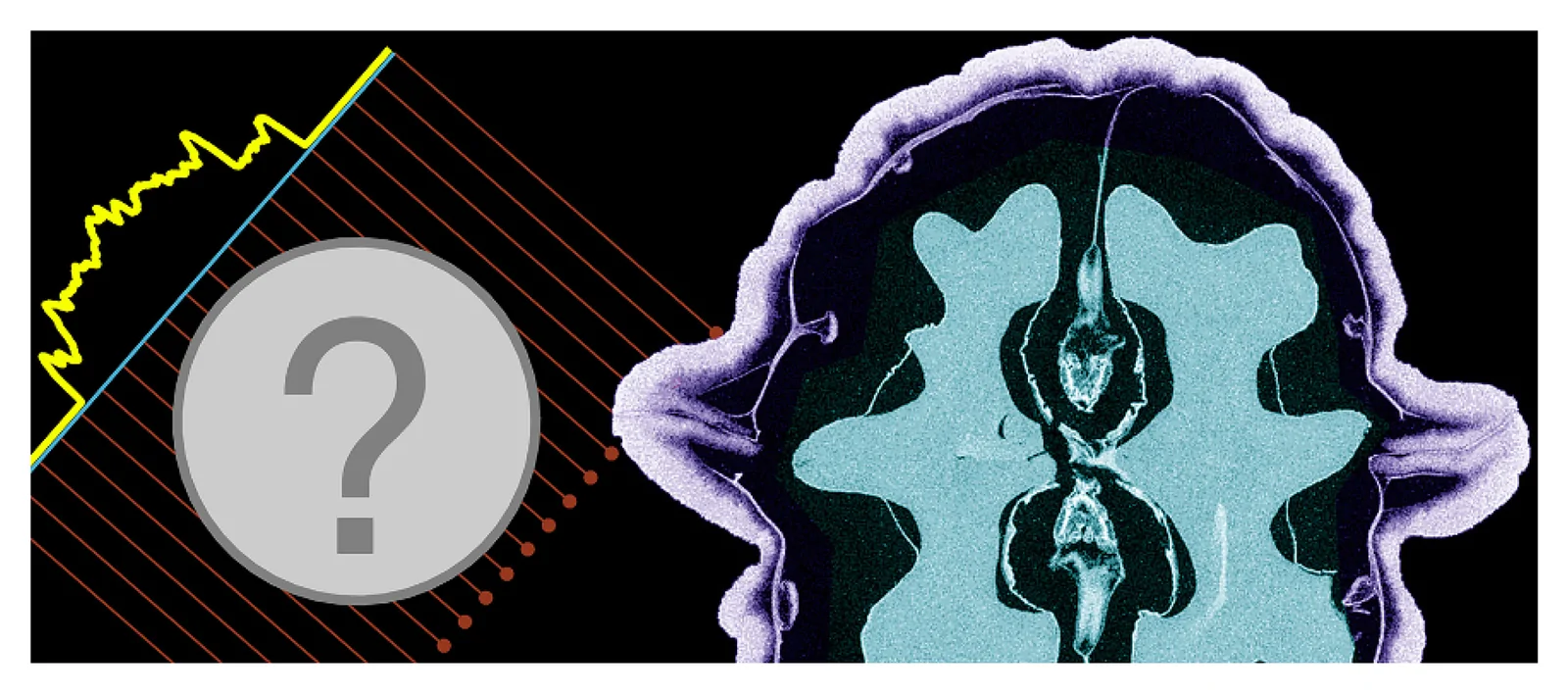
Demonstration of the principle of x-ray tomography. Measurements correspond to x-ray intensities detected after travelling through the imaged object along lines at different angles (left in the picture); the reconstructed image from such measurements is the linear attenuation coefficient of tissues. For an animated demonstration of x-ray tomography see www.youtube.com/watch?v=newxZbw7YAs. Reproduced figure with permission from Samuli Siltanen.

Once it is decided how to image, we face the task of turning the measurements into an image, cf figure 3. Apart from optical imaging where an image is formed by the device sensors, for all other medical imaging techniques such as CT, MRI, ultrasound, PAT, etc the image is indirectly captured (or hidden) in the measurements and an ‘inversion’ of the data is required before we can see an image. That is, measurements *f* and their relationship to the image *u* can be described by *f* = *A*(*u*) + *n*, where *A* is a linear or nonlinear so-called forward operator that models the measurement process, and *n* stands for random corruptions in the data. In CT, for instance, the detectors roughly speaking measure the intensity of the x-rays after having travelled through the body and being attenuated by the various tissues they pass through on the way. This constitutes the ‘forward problem’ of CT and mathematically can be modelled by *A* being the Radon transform. To see an image of the tissue attenuation *A* has to be inverted [75]. Another example is PAT, which belongs to the group of coupled-physics inverse problems. Here, the forward model *A* that connects the image to the measurements is given by a coupled system of partial differential equations (PDEs), one describing the optical part with a diffusion-type equation, and the other describing the acoustical part with a wave-type equation [6]. Such tomographic inversion is one of the main topics in the mathematical field of inverse problems, and is characterised by its usual ill-posedness due to the lack of a continuous inverse of *A*, incompleteness of and noise in the measurements. Often, there are multiple possible images that fit the measurements equally well. Robust solution strategies for inverse problems, therefore, involve careful physical modelling, functional analytic regularisation [17, 49] and/or statistical, in particular Bayesian, inversion models that can also provide uncertainty quantification [60, 93], applied harmonic analysis and compressed sensing [29, 41, 45, 69], and stable and scalable computational optimisation schemes [33]. Recently, also deep neural networks have been investigated for this task, with a clear outcome that such approaches can work very well in biomedical imaging context, but only in combination with physical models and thorough mathematical scrutiny [8], see also section 8. Also for image reconstruction, a close dialogue of researchers developing image reconstruction techniques and radiologists is important, see for instance this recently published online platform for MRI reconstruction [102] which aims to facilitate this collaboration.

## 4 From image to biomarkers

With increasing algorithmic capabilities, today, biomedical images are acquired not only for their qualitative analysis by a biomedical researcher or clinician who wants to study a disease or diagnose a patient, respectively, but for quantification purposes. Indeed, image analysis is a huge topic in healthcare analytics and—for it to be done at scale—requires accurate and fast automation procedures. Biomedical image analysis encompasses a number of mathematical areas: optimisation—as many image analysis tasks eventually amount to the solution of a large numerical optimisation problem; differential equations (in particular PDEs) and variational calculus [34, 88]—as derivatives of the image intensity function describe local changes and in particular image edges, and differential equations are frequently used for modelling of kinetics, while PDEs also underpin forward modelling of many nonlinear inverse problems e.g. electrical impedance tomography or ultrasound tomography; differential geometry and geometric measure theory [12, 21, 22]—as shapes of objects can be characterised by the length, curvature, tangent space etc of the object’s boundary such as in Chan–Vese segmentation [35] as exemplified in section 7, compare also (3); statistics and harmonic analysis—as multi-scale image structures can be represented by multi-scale function spaces e.g. wavelets [41, 69], or random fields [98]; functional data analysis [10, 81]—as image data is spatially and possibly time dependent, i.e. a function of position and possibly time, in contrast to most other types of data which are scalar; and machine learning—particularly deep neural networks that can be used to derive and represent data-driven image features trained on a large number of training examples [53]. Important image analysis and processing tasks that can be solved by one or more of the mathematical techniques above include

**Figure 4:**
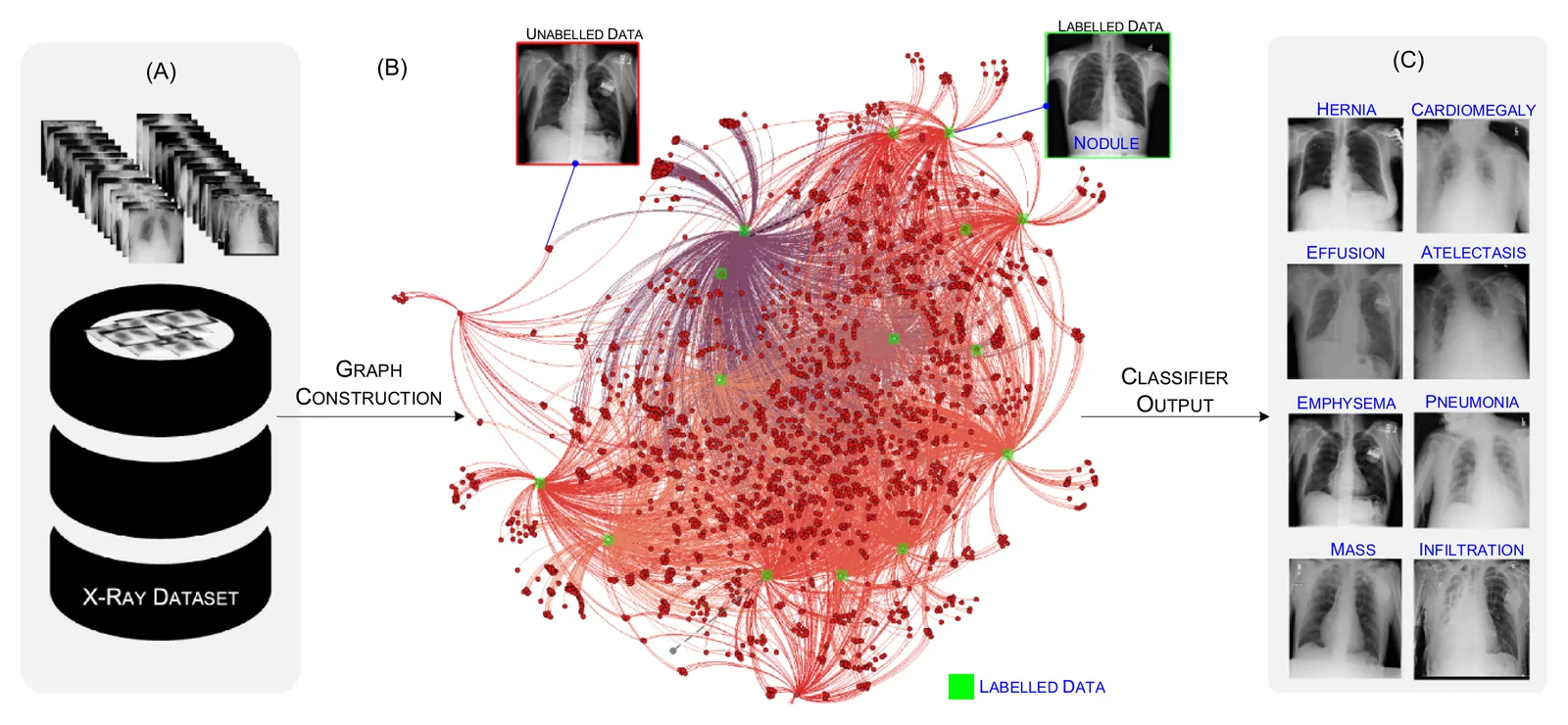
GraphXNet [14, 15]: graph classifier with minimal supervision for classifying chest x-rays into different pathologies.

(i) *Image segmentation* or *clustering* aims to segment one or more objects of interest in an image, also under the presence of noise and blur. A large community of researchers including mathematicians, engineers and computer scientists have investigated image segmentation, proposing and analysing models for this task. The methodologies used span a wide range, from machine learning to geometric measure theory [34, 35, 74, 86]. Segmentation tasks for biomedical imaging include delineation of lesions or individual organs in cancer therapy, for image-guided biopsies or generally for image-guided surgery [61, 72]. Compare also section 7 for a segmentation example using elements from geometric measure theory, embedded within a multi-tasking framework.
(ii) *Classification* is a specification of clustering, as it does not only create individual clusters but also assigns these with a class label. Classification can be done on a pixel-level—decomposing an image into different classes, e.g. cancerous tissue, margin, normal tissue—or an image-level—decomposing a dataset of images into different classes, e.g. tumour grades 1, 2 and 3. Classification is the fundamental principle of patient or disease stratification based on healthcare data. In the context of image-data, classification is done based on features derived from images. Deep neural networks constitute the state of the art for many biomedical classification tasks [50, 65], but also other machine learning methods such as support vector machines [16]. A major challenge for image classification algorithms is the usual lack of clean annotated data and hence classification methods with minimal supervision requirements, such as semi-supervised graph clustering & classification [14, 15] are being developed, cf also figure 4.
(iii) When acquiring images of an individual with different imaging modalities, e.g. MRI and PET, or at different times, or when comparing images from different individuals with each other, an important image processing task is the alignment of images and the fusion of information coming from different images. *Image alignment or image registration* [71], is particularly challenging for aligning images from different modalities as these can express very different features, making finding the correlations between them that can be used for the alignment difficult. Mathematical concepts involved here are based on differential geometry and non-convex optimisation. Once the images are registered the so-created multi-dimensional pixel array has to be processed further, e.g. feeding this into the segmentation or classification tasks from above. To do so, the appropriate *fusion of information from each image* is needed, which possibly involves a dimension reduction in the form of PCA or alike [51].
(iv) Bespoke *enhancement of images* in terms of contrast as well as in terms of resolution or for highlighting certain image features is another big topic in the image analysis part of the pipeline. This might involve customised filtering of the image, e.g. the edge enhancement can be realised via the classical Canny edge detector [31] as well as nonlinear PDEs [78], or scale-dependent filtering of images via novel spectral decompositions based on nonlinear eigenvalue problems [27]; or—the very timely and extremely difficult topic of—super-resolution or deconvolution for increasing spatial resolution of the image [44, 99].
(v) In the context of dynamic, or more generally spatio-temporal imaging, motion plays a major role, either as valuable additional information contained in the data or as a nuisance in which case it becomes a source of image artifacts. In the former case, *motion estimation* denotes the task of estimating velocity vectors for moving objects from a video-like sequence. Popular approaches include particle tracking (PIV) [80] and PDE-based approaches where the motion is modelled as a transport equation such as optical flow [11, 58], or recently also deep neural networks [46]. Motion features are important in biomedical imaging for the qualitative and quantitative analysis of, for instance, cardiac [13] or joint motion [43]. On the other hand, motion can also be a nuisance in tomographic imaging when interested in a static image which takes long enough to acquire so that e.g. respiratory motion cannot be suppressed via breath holding, or in functional imaging such as functional MRI [77]. Here, motion correction is crucial.

The outputs of the above processing steps are the source of new imaging biomarkers based on different imaging modalities and subsequent prediction, and the basis for image-guided biopsies, treatment and surgeries (e.g. which need automated detection and segmentation of objects from biomedical imaging data).

## 5 From image to answers

The final goal of the imaging enterprise can be a classification (stratification) of individuals into different groups for diagnosis, treatment recommendation or prediction in medical research or in the clinic, or target identification in drug discovery (here a drug target is meant as a protein or gene that interacts with, and whose activity is modulated by, a particular compound). This final step collates all the information that has been extracted from the imaging data in the previous steps and brings it forward to a statistical analysis, either on the imaging data itself, or on features extracted from the data. This leads to high-dimensional statistical problems [96] that require bespoke processing and analysis, e.g. sparse PCA [97] or high-dimensional classification [30], and statistical analysis of longitudinal (time-dependent) data [40, 56].

Let us now highlight three exemplar case studies in biomedical imaging research that touch upon recent developments and offer perspectives towards future research trends in this field.

## 6 Multi-modal image reconstruction

6MRI is a highly customisable modality with a number of contrasts routinely measured in clinical practice. Among the most popular are the so called T1, T2 images obtained by weighting with the corresponding relaxations times while other common contrasts include fluid-suppressed images (FLAIR), diffusion and susceptibility weighted images and post-contrast images revealing function.

However, by the nature of sequential **k**-space (Fourier domain) sampling, each MRI scan is relatively slow to acquire, the scan time depending on the size of the region being scanned. The main bottle neck is caused by the magnetic gradients used for selecting the point in the **k**-space, which cannot change indiscriminately fast due to physical and physiological constraints. As a consequence, while many properties of the tissue can be measured, there is a trade-off to be made between the scanning time, which is directly related to the image resolution, but also potential data deterioration due to e.g. motion if the patient cannot remain completely still over such a long period of time.

An analogous problem arises in dual/multi- energy x-ray CT. The presence of two images at different energy levels allows to untangle the attenuation coefficient into atomic number and density [4] facilitating material decomposition. In most scanners this comes at a cost of higher dose of ionising radiation delivered to the patient due to acquisition of low and high *keV* images which is a primary concern. The recent development of the photon counting detectors enables multi-energy (also called spectral or colour) x-ray imaging without need of multiple irradiation via a posterior binning of the energies of the collected photons. An example of such a detector is Medipix3/Medipix4 technology developed at CERN for high resolution, multi-energy and low noise imagery which made its way into medical imaging^4^.

The above examples have in common that each of the contrasts can be measured essentially with the same resolution. Another category are problems where one contrast can be acquired with higher resolution than the other, examples include PET-MRI, SPECT-CT. In both cases however, the underlying anatomy is the same which underpins the mathematical approach to multi-modal reconstruction.

**Figure 5:**
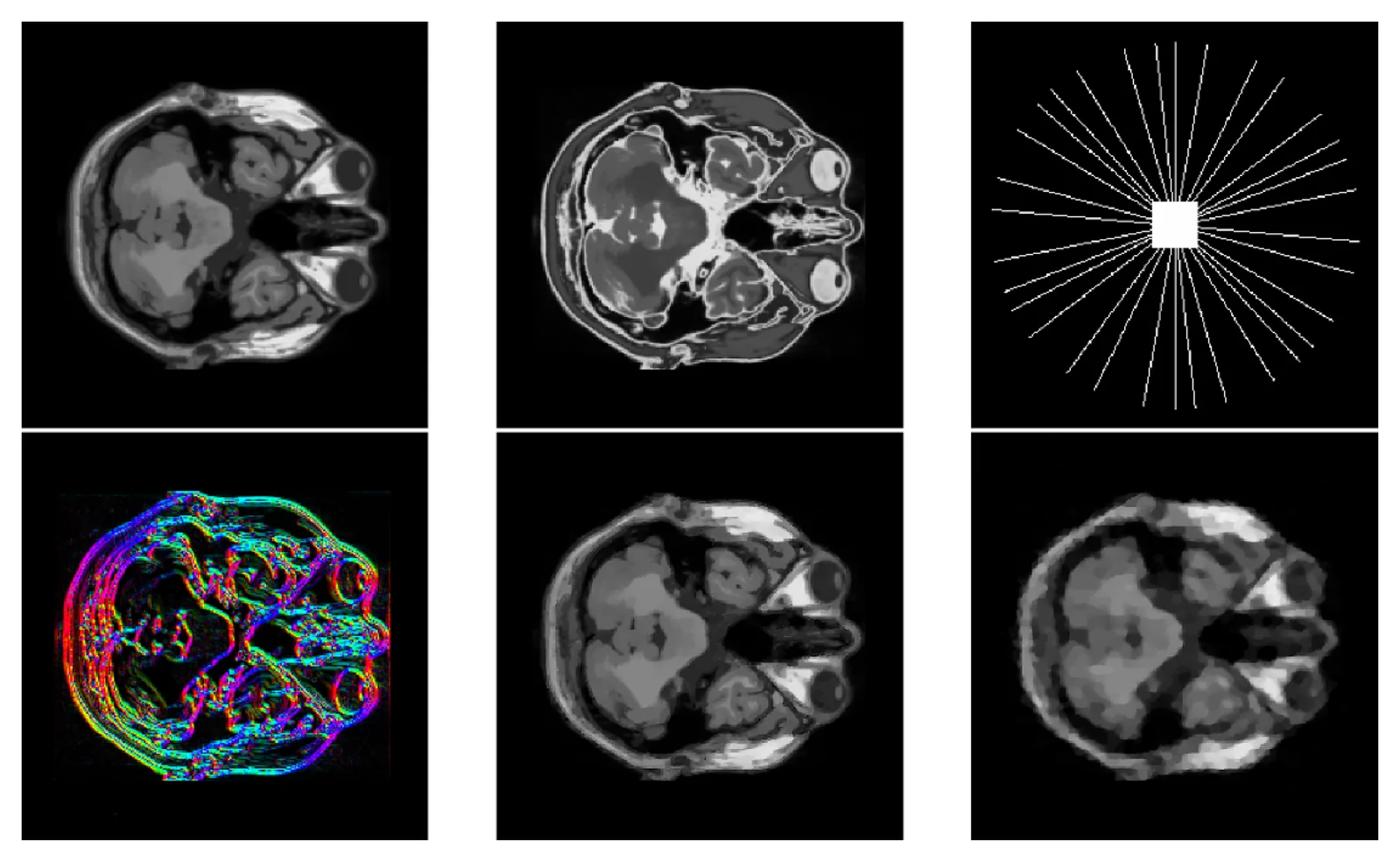
Demonstration of the importance of exploiting the coherences between contrasts on an example of MRI: reconstruction of subsampled T1 given full data reconstruction of T2 [47]. Top row: T1, T2 images (original source: BrainWeb [37]), subsampling scheme applied to T1. Bottom row: direction of the edges derived from T2, T1 reconstruction exploiting T1 & T2 coherence via directional total variation regularisation with edge directions derived from T2 image, T1 reconstruction agnostic to T1 & T2 coherence using isotropic total variation regularisation. Reproduced from [47]. CC BY 4.0.

Such joint reconstruction of multiple contrasts based on an assumption of high correlation between the images originating from the same object have recently attracted attention of the inverse problems community with the goal to obtain same quality reconstruction from an, per contrast, incomplete set of measurements. Many joint reconstruction algorithms make a supposition that the multiple contrast images share a common (sub)set of edges which is motivated by the underlying anatomic features. This strategy underpins the parallel level set approach developed in [48] relying on smooth optimisation formulation and the directional total variation approach proposed in [47] cf figure 5, which is essentially a non-smooth convex analog to the parallel level sets. These approaches result in a biconvex optimisation problem for the joint reconstruction formulation, i.e. only convex if considered as a reconstruction of one unknown contrast *u* : Ω → ℝ (where Ω is a rectangular grid of image pixels) informed by another known contrast *v* : Ω → ℝ. Such structure guided convex reconstruction problem can be mathematically formulated as

(1) $$u=\underset{u⩾0}{\arg\min}\frac{1}{2}\|Au-f{\|}_{2}^{2}+\alpha {\text{dTV}}_{v}(u),$$

where *A* is the subsampled Fourier transform, *f* the corresponding noisy undersampled **k**-space data and the regularisation parameter *α* > 0 strikes the balance between the data fit and regularisation, here the directional total variation derived from known contrast *v* = (*v*_*n*_)_*n*∈Ω_ is defined as

(2) $${\text{dTV}}_{v}(u):=\sum_{n\in \text{Ω}}|{𝒫}_{\xi_{n}}\nabla u_{n}|,\;{𝒫}_{\xi_{n}}x:=x-\langle \xi_{n},x\rangle \xi_{n}$$

with the structure encoded using the vectorfield

$$\xi_{n}=\frac{\nabla v_{n}}{\sqrt{{\left|\nabla v_{n}\right|}^{2}+\eta^{2}}}:\;0⩽\left|\xi_{n}\right|<1,\;\eta >0.$$

See here for the associated code and testdata https://github.com/mehrhardt/Multi-Modality-Imaging-with-Structure-Promoting-Regularizers

Another family of approaches is based on total nuclear norm minimisation proposed in e.g. [57] and extended to multi-energy x-ray CT in [83] which results in a convex problem for the joint reconstruction.

At this point we would like to take note of potential dependencies generated by considering a joint (as opposed to separate) reconstruction problem onto the imaging pipeline at large:

(i) *Backward*: the optimal set of measurements to acquire for each of the contrasts depends on the assumed coherence such as e.g. assumption of the common set of singularities;
(ii) *Forward*: the assumed coherence should be transparently fed into the following image/statistical analysis and assisted or automated prognosis stages.

## 7 Multi-tasking in biomedical imaging

Each of the stages of the biomedical pipeline in figure 1 is usually completed and the results fed into the next. This modularity is a result or artefact of how the pipeline has historically developed and has many advantages: each stage has defined familiar outputs and it can be independently declared success or failure according to some established criteria, each stage of the pipeline can be altered without impacting the others, methodology for each stage can therefore be independently developed by different research communities with minimal communication overheads. However, at each stage independent potentially unaccounted for in the following stages or even conflicting assumptions are being made which can, if not deteriorate the final result, then at least render the results sub-optimal and make their rigour hard to argue.

With the advances constantly being made throughout the stages of the pipeline, each individual stage is becoming more and more customisable calling the validity of the present solution increasingly into question and demanding alternatives.

In view of this, there are several attempts towards linking up these individual steps and design a holistic, all-in-one approach for biomedical imaging. The idea of such an approach is to make use of the full information in the measured data in every step of the analysis pipeline, e.g. in the image reconstruction, the segmentation, classification, prediction etc very much in contrast to the sequential approach which can only optimise each step independently of the next resulting in possible biases, carried forward in each step, and sub-optimal solutions overall.

Some examples of such approaches are task-adapted reconstruction with deep learning [2], motion-enhanced reconstruction [25, 26], joint reconstruction and motion correction [38], joint reconstruction and segmentation [28, 39, 62, 92] and joint reconstruction and classification [70]. In [39], for instance, the authors consider a non-smooth and non-convex optimisation approach for joint reconstruction and segmentation from MRI data. Mathematically, given the under-sampled and noisy **k**-space data *f*, we want to recover the image *u*: Ω → ℝ (where Ω is a rectangular grid of image pixels) and compute its segmentation *v* in 𝓁 disjoint regions, by solving the following problem

(4) $$(u,v)=\underset{u,v}{\arg\min}\underset{\text{reconstruction}\;}{\underset{︸}{\frac{1}{2}\|Au-f{\|}_{2}^{2}+\alpha \text{TV}(u)}}+\underset{\text{segmentation}\;}{\underset{︸}{\delta \sum_{i=1}^{n}\sum_{j=1}^{\ell }v_{ij}{\left(c_{j}-u_{i}\right)}^{2}+\beta \text{TV}(v)+ıC(v)}},$$

where *A* is the subsampled discrete Fourier transform, and *𝒾*_𝒸_(*v*) is the characteristic function over $C:=\left\{v:{\mathbb{R}}^{n}\to {\mathbb{R}}^{\ell }|v_{ij}⩾0,\sum_{j=1}^{\ell }v_{ij}=1,\forall i\in \{1,\ldots ,n\}\right\}$ The total variation (TV) regularisation is a well-known edge-preserving smoothing functional for images, first introduced by Rudin, Osher and Fatemi in [87] for image denoising. The TV regularisation is the 1-norm penalty on a discrete finite difference approximation of the two-dimensional gradient ∇ : ℝ^*n*^ → (ℝ^2^)^*n*^, that is ∇*u*(*i, j*) = (∇_1_*u*(*i, j*), ∇_2_*u*(*i, j*))^*T*^, i.e.

$$\text{TV}(u)=\|\nabla u{\|}_{2,1}=\underset{(i,j)\in \text{Ω}}{\sum }\sqrt{{\left|\nabla_{1}u(i,j)\right|}^{2}+{\left|\nabla_{2}u(i,j)\right|}^{2}}.$$

**Figure 6:**
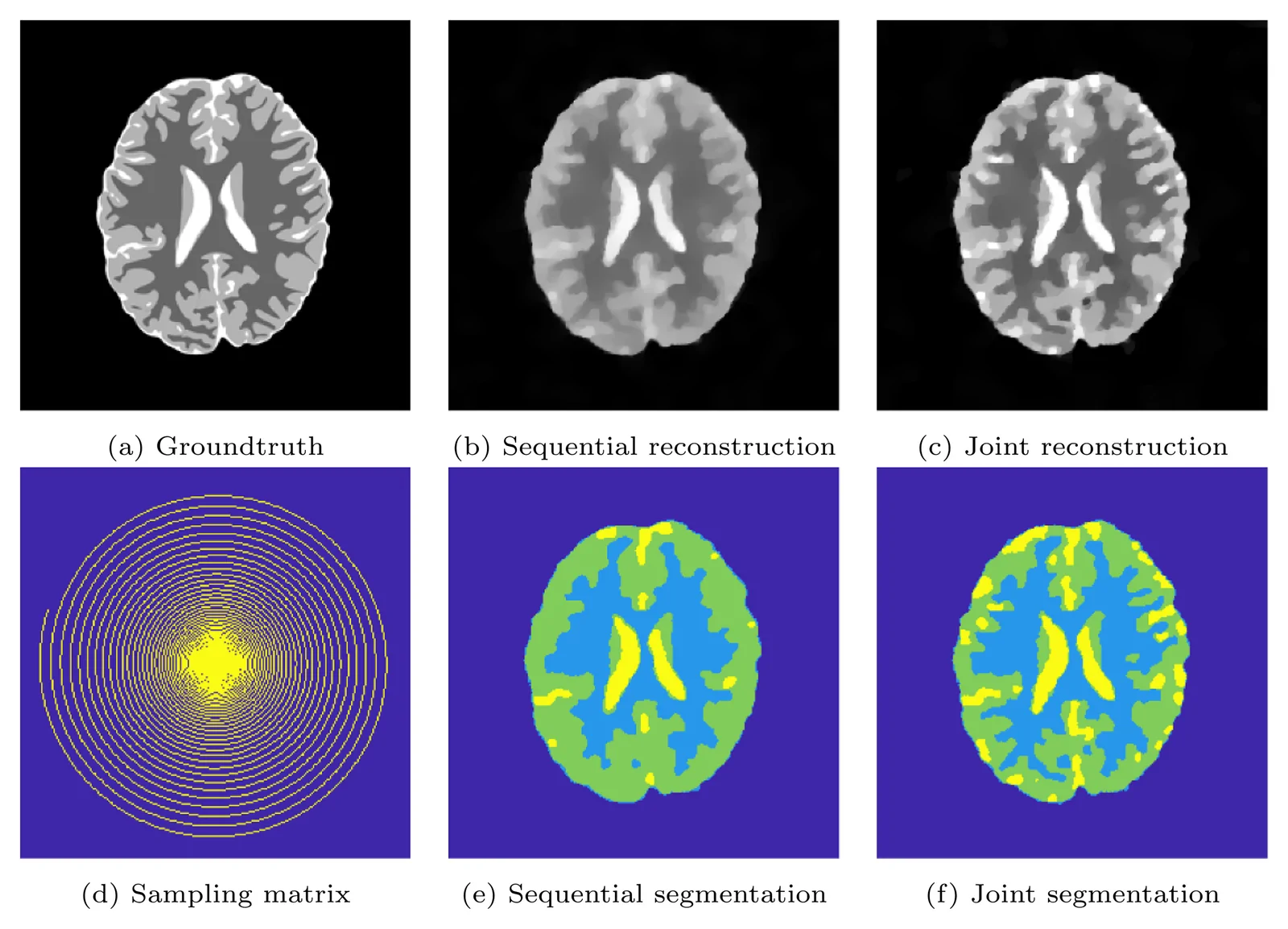
Sequential approach (centre) versus unified approach from [39] (right). Combining reconstruction and segmentation in a single unified approach improves both the reconstructed image and its segmentation. Reproduced from [39]. © IOP Publishing Ltd. CCBY3.0.

Moreover, the segmentation in (3) is modelled using the so-called Chan-Vese approach [35] which is a special case of the Mumford–Shah model [74]. The parameters *α, β*,*δ* > 0 determine the relative weighting of the functionals in the optimisation problem. Figure 6 demonstrates the effectiveness of the joint reconstruction and segmentation model in (3) in comparison with a sequential approach of first reconstructing an image and subsequently segmenting it. Comparing the resulting segmentations with the ground-truth, the superiority of the joint solution pipeline is visible. See here https://github.com/veronicacorona/JointReconstructionSegmentation for the implementation and exemplar test data.

The bottleneck of approaches such as (3), however is the non-smoothness coupled with the non-convexity of jointly solving the tasks, and the dependence of minimisers on the choice of the free positive parameters *α, β* and *δ*. The latter can be handled through parameter choice rules such as the discrepancy principle [73] or through bilevel optimisation [42]. The non-smoothness and non-convexity are inherent to the approach and cannot be easily remedied. A promising direction for lifting this computational bottleneck is the idea of combining the idea of multi-task learning [32] with deep neural networks [2], which—once trained—are just a concatenation of a relatively small number of explicit operations applied to the input data.

## 8 Deep learning revolution in biomedical imaging

The deep learning revolution also had a huge impact on biomedical imaging and biomedical image analysis. Deep learning is strongly based in mathematics as neural networks are nothing but a concatenation of a finite number of linear and nonlinear operations, and their training amounts to the solution of large-scale non-convex optimisation problems. Indeed, see here for an overview of the mathematics of deep learning [54]. Many success stories appeared in the literature that were reporting results to biomedical image analysis tasks which were previously unthinkable. For example, in a collaboration between DeepMind, Moorfields Eye Hospital and Google Health researchers have developed a deep learning model that when fed with OCT imaging data of the retina could predict the development of exudative age-related macular degeneration (exAMD), a serious form of age-related macular degeneration. They demonstrated that their system is able to perform as well as, or better than, clinicians at predicting whether an eye will convert to exAMD in the next 6 months [101]. Another example is the effect of deep learning on medical image reconstruction as introduced in section 3. Here, in the presence of appropriate training data, deep learning has quickly overtaken more classical approaches such as variational models from section 6. Tying in with the mathematical approaches seen so far a relatively natural extension featuring deep learning, is the idea of replacing a handcrafted regularisation such as (2) by a regularisation that is parametrised by a deep neural network and optimised with appropriate training data [64, 67]. The idea in [67], for example, is to train a neural network regulariser in an adversarial fashion. More precisely, the adversarial learning framework trains the regulariser to discriminate between a distribution of ground-truth images *π*_1_ and a distribution of baseline reconstructions *π*_2_ using the following loss function

$$\underset{ℛ\in \;l-\text{Lipschitz}\;}{\min}E_{X\sim \pi_{1}}ℛ(X)-E_{X\sim \pi_{2}}ℛ(X).$$

**Figure 7:**
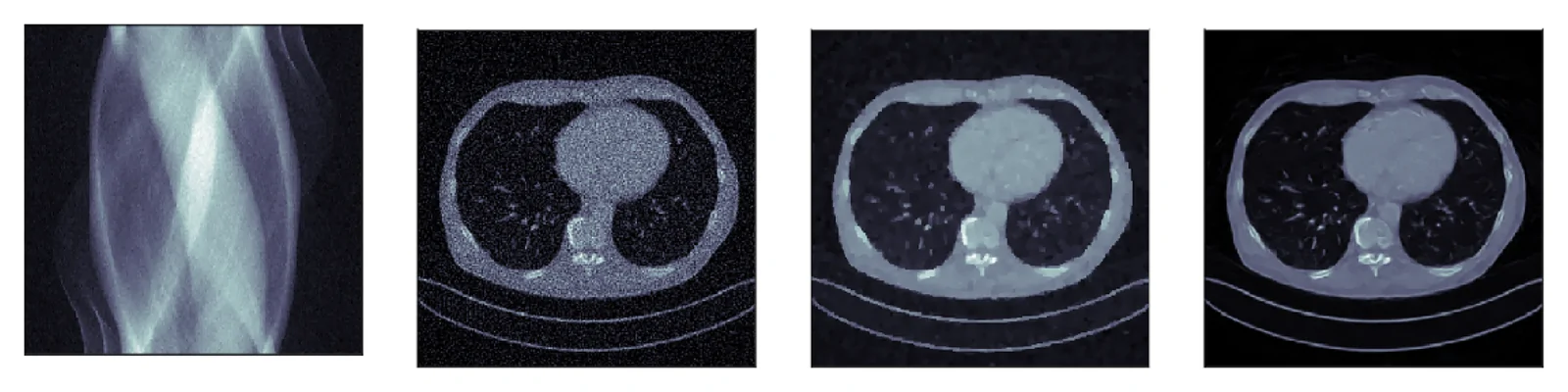
(fltr) Filtered backprojection reconstruction, Reconstruction with total variation regularisation similar to (1); reconstruction with a deep neural network regularisation [67].

Using the resulting regulariser ℛ∗ for reconstructing an image from sparse angle and low-dose CT measurements, gives the result in figure 7. See here for downloadable code for a convex variant of the adversarial regulariser https://github.com/Subhadip-1/data_driven_convex_regularization. Neural network regularisers are only one example of how deep learning entered biomedical image reconstruction approaches. Others are, for example, learned post-processing [59], learned unrolling (or learned iterative schemes) [3, 52, 55] or plug-and-play regularisation [85, 95], see also [8] for a recent review article on the topic.

From these examples is seems that, with only the right training data—which usually means enough high-quality training examples—deep learning offers a magic key into problems that we considered almost unsolvable.

As time progressed, however, also the challenges and quite critical hurdles of translating deep learning into biomedical imaging practice became clear. For the claim of George Hinton in 2016 that ‘Radiologists would be out of a job in 5–10 years’, to become true there are many more problems for machine learning and biomedical imaging researchers to overcome.

One of these certainly is the need for more mathematical guarantees for deep learning based image analysis and processing. While perhaps it is natural that mathematical theory needs to catch-up with numerical evidence for new methodological paradigms such as deep learning, it is also crucial for delivering efficient, safe and interpretable AI tools for biomedical imaging. We are definitely seeing many advances in this direction, with more general mathematical theory for deep learning being developed [54] and in particular for biomedical imaging [8], but there are still many more miles to go when compared to the mathematical foundations of more established approaches discussed earlier.

Another big hurdle that needs to be overcome before a large roll-out of these tools can happen is their dependence on large and high-quality training data or in other words, the inability of deep learning based approaches to generalise well from one dataset to another. In particular, while deep neural networks generalise surprisingly well to unseen images from the same underlying distribution (e.g. MRIs of healthy individuals, imaging the same field of view, all acquired in the same way by the same scanner manufacturer), they are struggling when applied to out-of-distribution images (e.g. a neural network trained on MRIs of healthy knees applied to MRIs of knees suffering from osteoarthritis, or trained on MRIs acquired by Siemens and applied to MRIs acquired by General Electric, etc). This stands in stark contrast with medical practice where datasets of biomedical images are usually moderately sized and where sharing of data still requires significant effort. The COVID pandemic has just showcased this and several other issues related to the development and deployment of AI tools for healthcare and biomedical image analysis in particular [84]. It is hard to give exact numbers for required dataset sizes here because they depend on many factors such as heterogeneity of the underlying data distribution. From experience, however, most available biomedical imaging datasets right now are orders of magnitude too small for the requirements of deep learning algorithms. This will certainly change, and it is changing, with increasing efforts in collaborative data curation between institutions and hospitals, such as UK Biobank [19], as well as, on the machine learning end, promising developments of semi-supervised, weakly supervised, and un- and self-supervised deep learning approaches for medical imaging, see for instance [66], and interesting new research into the simulation of synthetic training data [100], but there is still way to go.

## 9 The way ahead

Biomedical imaging offers a myriad of opportunities for mathematicians to contribute. All of the stages of the pipeline are lined with beautiful mathematical theory and methodology, each with its own challenges and varying mathematical principles involved. The provision of AI methods that offer an automated and robust analysis of imaging data on a large scale, and integrating them with non-imaging data such as patient demographics, blood biomarkers and genomics, is what is needed for optimising healthcare procedures and making them more personalised. Developing such purposeful AI tools for clinical practice requires a close collaboration between mathematicians, statisticians, computer scientists, biomedical researchers and clinicians. The success of deep neural networks as current state-of-the-art techniques for many of the tasks we encountered along the biomedical imaging pathway seems to render them ideal candidates such AI healthcare methods should be based on. To prepare them for their application to sensitive decision making in the clinic, however, the gap between the empirically demonstrated deep learning capabilities and their lack of robustness and explainability needs to be closed first, through rigorous mathematical treatment of these techniques.

## Data Availability

All data that support the findings of this study are included within the article (and any supplementary files).

## Footnotes

3 www.ibmbigdatahub.com/infographic/four-vs-big-data.

4 https://medipix.web.cern.ch.
